# An exploration of the tractability of the objectivist frame of disaster risk in policy implementation in Zimbabwe

**DOI:** 10.4102/jamba.v11i1.604

**Published:** 2019-05-21

**Authors:** Paul Chipangura, Dewald van Niekerk, Gerrit van der Waldt

**Affiliations:** 1 Unit for Environment Sciences and Management, African Centre for Disaster Studies, North-West University, Potchefstroom, South Africa; 2 Institute of Development Studies, Bulawayo National University of Science and Technology, Bulawayo, Zimbabwe; 3 Faculty of Humanities, Social Transformation, North-West University, Potchefstroom, South Africa

**Keywords:** objectivist frame, disaster risk, tractability, policy, implementation

## Abstract

Despite the growing evidence pointing towards disaster risk as a social construction, the objectivist frame still dominates the conceptual frameworks constructed around disaster risk in Zimbabwe. As disasters continue to occur with increasing regularity and ferocity, the usefulness of the objectivist frame of disaster risk in minimising the devastating effects of disasters is questionable. This article investigates how framing affects the tractability of the objectivist frame of disaster risk in Zimbabwe by using the Tokwe-Mukosi flood disaster of 2014 as a case study. The research utilised secondary data and semi-structured interviews with senior managers and specialists in disaster risk management in Zimbabwe to explore factors affecting the tractability of the objectivist frame in implementation. The results of the study suggest that tractability of the objectivist frame is mainly affected by its limited understanding of the causes of, and solutions to, disasters. The frame ignores rival frames crucial in disaster causality, such as the constructivist frame, and in ‘ignorance’ it harbours ‘latent’ failures which only become apparent on the occurrence of a particular major disaster. Moreover, the objectivist frame of disaster risk requires significant administrative and technical expertise and funding to be tackled effectively, which are not readily available especially in developing countries. The frame is also reactive in dealing with disasters, which makes it prone to ‘policy surprises’, leading to ‘policy disasters’ where disasters are viewed as direct consequences of policy choices. The article concludes that for Zimbabwe to achieve its goal of minimising the impacts of disasters, greater efforts must be made in reframing disaster risk by integrating the objectivist frame with the social constructivist frame.

## Introduction

Despite the growing evidence pointing towards disaster risk as a social construction, the objectivist frame of disaster risk still dominates the conceptual frameworks and imageries constructed around disaster risk in Zimbabwe (Chipangura, Van Niekerk & Van Der Waldt 2017; Lavell & Maskrey 2013). This view of disaster has inspired interpretations of what disaster risk means and controls how certain phrases are used, prioritises the questions that are asked and answered, and influences the solutions that are prescribed (Leichenko & O’Brien 2008) for disaster risk. Consequently, the emphasis on managing ‘natural’ disasters has become conventional wisdom and is locked into policies, governance arrangements and instrumental systems (Lavell & Maskrey 2013) such as the *Civil Protection Act* in Zimbabwe (Chipangura et al. 2017). However, with policies that are informed by the objectivist frame, both anthropogenic and natural hazards continue to occur with increasing regularity and ferocity, killing hundreds of people and destroying millions of dollars of habitat and property in Zimbabwe (EM-DAT 2016). This implies that such policies do not often lead to concrete measures that reduce disaster risk and the devastating impacts of disasters. In this respect, they seem to resemble ‘empty shells’ (Hoque & Noon 2004) characterised by ‘implementation deficit’ (Knoepfel et al. 2011). As a result, policy failures have tended to produce calls for more regulation, with little assessment of the underlying reasons for failure (Lavell & Maskrey 2013; OECD 2000).

Studies of disaster risk governance suggest that the way disaster risk is framed underlies many policy implementation failures (Handmer & Dovers 2013; Merry 2013; Renn 2008). This is because framing is responsible for the emergence of problems, for the way in which they are seen, for the way in which they are approached and considered, for the kind of remedial plan that is laid out and for the transformation of the remedial (Handmer & Dovers 2013; Grafton & Permaloff 2005). Framing therefore determines the regulatory path and how successfully the chosen management strategy is implemented (Merry 2013). However, research on framing has largely focused on the level of policymaking, analysing how policy entrepreneurs and policymakers define problems and embed them in public policy (Coburn 2006). In disaster risk literature, little, if any, attention has been given to what the objectivist frame means for disaster risk policy designs, and with what, consequences for their implementation. This article therefore seeks to contribute in filling this gap by investigating how framing affects the tractability of the objectivist frame of disaster risk, which dominates disaster risk practice in Zimbabwe in minimising the devastating impacts of disasters. The article illustrates the practical consequences that the objectivist frame has for disaster risk reduction policy and responses by drawing on Tokwe-Mukosi flood disaster that occurred in Zimbabwe in 2014.

### Framing

Disaster risk has been understood differently by different scholars and practitioners depending on a range of factors, such as philosophical orientation, values, beliefs and the institutional context in which disaster risk reduction is planned or implemented. It has been viewed as acts of God, act of nature and as socially constructed events (Chipangura, Van Niekerk & Van Der Waldt 2016; Quarantelli 2005). The process by which issues, decisions or events acquire different meanings from different perspectives has been studied as ‘framing’ in a variety of social science disciplines (Entman 1993; Snow 2004). To frame is to select some aspects of perceived reality and make them more salient in the communicating text, in such a way that it can promote a particular problem definition, causal interpretation, moral evaluation and/or treatment recommendation for the item described (Hallahan 1999). Authors such as Handmer and Dovers (2013) and Grafton and Permaloff (2005) argue that framing influences how a problem is defined and constructed as well as how the governance arrangements, incentives and instrumental systems developed to address the problem are designed. Framing has been shown to affect people’s decision preferences, particularly under conditions of uncertainty (Tversky & Kahneman 1981). Problems that are formulated in different ways trigger different preferences. For example, Dewulf (2013:322) argues that, when people are asked whether they would favour a particular case of dike enlargement, one can expect a much higher percentage of positive answers if the question was prefaced with ‘given the importance of long-term flood safety’, compared to the preface ‘given the importance of the rights of the current property owners that would have to move’. Framing can be categorised into diagnostic or prognostic (Snow & Benford 1988). Diagnostic framing generally refers to the identification of a problem and who to blame (Snow 2004). According to Cress and Snow (2000), diagnostic framing is important because particular problem definitions are advocated while others are undermined. Prognostic framing is about the identification of a solution to the problem identified in the diagnostic framing (Snow 2004). In articulating a proposed solution, prognostic framing sets forth particular goals and suggests tactics for achieving those goals (Snow & Benford 1988). Prognostic and diagnostic framings may be challenged as others offer counter-frames that put forth alternative portrayals of the situation, often with contrasting implications for roles, responsibility and resources (Fligstein 2001). In understanding how framing affects policy implementation, this article focuses on the objectivist frame of disaster risk, which has dominated the ontological and epistemological viewpoints in disaster risk research and practice.

### The objectivist frame of disaster risk

Objectivism is based on the belief that there is an objective reality and that knowledge exists as something that can be observed and measured (Chipangura et al. 2016). The social world can thus be known epistemologically through the use of objective instruments of measurement operated by a rational and neutral researcher (Eriksson & Kovalainen 2008). From an objectivist view, disaster risk is understood as a physical phenomenon that can be objectively assessed (Chipangura et al. 2016). According to Cardona (2003), this view has been largely shaped by geophysicists, seismologists, meteorologists, geologists and epidemiologists, among others, who believe that disaster risk is a topic exclusively associated with the physical phenomena that generate natural hazard or disaster events and can be objectively assessed. Disaster risk can therefore be understood as a probability of loss in relation to the impact of a specific hazard (Shefali 2009). As Chipangura et al. (2017) argue, this view of disaster risk is hazard centric in that it places disaster risk problem on the hazard; hence, it is generally referred to as the hazard paradigm. This conjecture that disasters only result from natural forces is tantamount to saying that they lie solely outside human history, beyond human influence, beyond moral reason and beyond control (Steinberg 2006).

The hazard paradigm aims to reduce disasters through control of natural environment (Lowe et al. 2007). This is premised on the assumption that: ‘it is possible to manage the planet if there is sufficient knowledge of all the interactions in such large physical systems as the atmosphere, hydrosphere, lithosphere, atmosphere, and biosphere’ (Wisner et al. 2004). According to Lowe et al. (2007), the hazard approach seeks to manage risk through public policy application of geophysical and engineering knowledge, for example, monitoring and modelling extreme geophysical events, and creating disaster and emergency plans. However, this view has been criticised in that not all disaster risk problems can be solved using the application of geophysical and engineering knowledge. According to Pelling (2003), technological responses that deal with physical causes alone can prolong, and even increase, the losses incurred when disasters occur. Moreover, discourse of disaster causality tends to overlook the socio-economic processes that place the vulnerable populations at risk, and as a result, such processes are not considered as policy issues. Humanity is viewed as powerless victims of ‘natural’ disasters (Jones & Murphy 2009), and thus free from the responsibility of avoiding disasters. Disaster risk policies that emanated from the objectivist frame have however been difficult to implement because they have been geared towards addressing the hazard component only, yet disaster risk is a product of the possible damage caused by a hazard because of the vulnerability within a community (Van Niekerk 2012).

### Factors affecting policy implementation

Public policy may be viewed as a purposive course of action taken by governments in dealing with some problems. According to Handmer and Dovers (2013), policies are positions taken and communicated by governments in more or less detail ‘avowals of intent’ that recognise a problem and state what will be done about it. These definitions show that public policies do not just happen haphazardly. Instead, public policy involves the production of deliberated decisions or (non)actions that involve public actors and the intention to solve what is perceived as one or several collective problems (Knoepfel et al. 2011). The movement from policy on paper to action on the ground has been studied by scholars as policy implementation, which aimed to explain causal relationships between policies as enacted and policies as implemented (Koontz & Newig 2014). Policy implementation, as Brynard (2007) argues, is concerned with the following questions: were the intentions of the policy translated into tangible outputs? Did the outcomes of the policy match its goals? What is being implemented? How is policymaking differentiated from policy implementation? Policy implementation thus identifies the problem(s) to be addressed, stipulates the objective(s) to be pursued and, in a variety of ways, ‘structures’ the implementation process. In trying to understand the factors that affect implementation, Sabatier and Mazmanian (1980) present three categories of factors thought to affect the implementation of public policy in their framework. This study utilises two categories: (1) tractability of the problem and (2) the ability of the statute to structure implementation, which are explained below.

#### Tractability of the problem

It concerns the political, societal and technical capacity of managing and taming a collective problem through proposed policy solutions (Hisschemöller & Hoppe 1995). According to Dovers (1995), tractability describes the ease, or conversely the difficulty, with which a problem can be redressed. Sabatier and Mazmanian (1980) argue that problems are most tractable if:

(1) there is a valid theory connecting behavioural change to problem amelioration; the requisite technology exists; and, measurement of change in the seriousness of the problem is inexpensive; (2) there is minimal variation in the behavioural practices which cause the problem; (3) the target group constitutes an easily identifiable minority of the population within a political jurisdiction; and (4) the amount of behavioural change is modest. (p. 542)

#### The ability of the statute to structure implementation

According to Sabatier and Mazmanian (1980), the ability of the statute to structure implementation is built around the view that policymakers can substantially affect the attainment of legal objectives by utilising the levers of power at their disposal to coherently structure the implementation process. They argue that legislation that seeks to significantly change target group behaviour in order to achieve its objectives is most likely to succeed if:

(1) it incorporates a valid causal theory linking behavioural change to desired impacts; (2) its objectives are precise and clearly ranked; (3) it provides adequate funds to the implementing agencies; (4) the number of veto points in the implementation process is minimized and sanctions/inducements are provided to overcome resistance; (5) the decision rules of the implementing agencies are biased toward the achievement of statutory objectives; (6) implementation is assigned to agencies which support the legislation’s objectives and will give the program high priority; and (7) the provisions for outsider participation are similarly biased through liberalized rules of standing and by centralizing oversight in the hands of statutory supporters. (p. 542)

## Methodology

For purposes of analysis, this study is approached from an interpretivist perspective. This perspective provides an opportunity to understand the way people interpret and make sense of their experiences in the world in which they live and how the context of events and situations and the placement of these within wider social environments have impacted the constructed understandings and realities (Crotty 1998). This study therefore used discourse and document analysis together with qualitative semi-structured interviews in order to investigate how framing affects the tractability of the objectivist frame of disaster risk, which dominates disaster risk practice in Zimbabwe, using Tokwe-Mukosi flood disaster as a case study.

### Case selection

**FIGURE 1:**
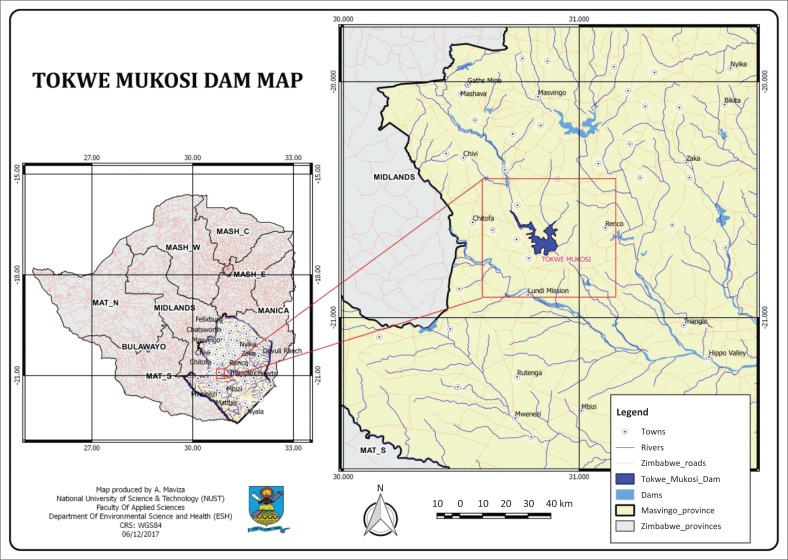
Location of Tokwe-Mukosi dam.

Tokwe-Mukosi dam is situated in the semi-arid area of Southern Masvingo region (Figure 1). The Tokwe-Mukosi flood disaster that occurred in early February 2014 was selected because it is one of the worst flood disasters that had occurred in Zimbabwe. According to the Department of Civil Protection (2014), about 2500 households upstream of the Tokwe-Mukosi dam were displaced to Chingwizi, Chisase and Masangula relocation sites of Nuanetsi ranch in Mwenezi district. The Department of Civil Protection further noted that the majority of these households were mainly low-income families. The incomplete dam partially collapsed and induced flooding to about 40 000 people downstream. It was believed that the dam would fill up by December 2015 when all the communities living within the dam basin would have been relocated to designated sites (Department of Civil Protection 2014). The 6393 households (approximately 32 000 people) and 18 764 cattle were supposed to be relocated in three phases. Phase 1 intended to relocate about 1247 households in very low-lying areas of the dam basin. These households were supposed to be relocated by October 2013. Phase 2 intended to relocate a further 1878 households by October 2014. Finally, the last phase would relocate 3268 families in hinterland areas by October 2015 before the dam was filled up. However, flooding occurred when the dam was incomplete and only 600 households had been relocated (Department of Civil Protection 2014). The remaining 5793 households were caught by surprise in the unsafe basin. The government made an appeal to both local and international humanitarian communities to aid urgent humanitarian needs for affected communities in the Tokwe-Mukosi Dam Basin (Department of Civil Protection 2014).

### Data collection and analysis

This study utilised secondary data and semi-structured interviews. For secondary data collection, most of the documents were collected through electronic databases. Libraries in Zimbabwe and search engines like Google Scholar were also utilised. Different search combinations were used to find relevant documents. ‘Policy’ was combined with words like ‘implementation’, ‘framing’, ‘tractability’ and ‘disaster risk’. A critical evaluation of the credibility of the documents was done mainly by cross-examining different authors’ logic of argumentation and conclusions. Documentary analysis and discourse analysis (DA) were used to analyse the vocabulary used to describe disaster risk in books, policy documents and research documents. The empirical part of the research used semi-structured interviews to explore the tractability of policy frames in Zimbabwe. The interviews were designed to collect information on the factors affecting tractability of the objectivist frame of disaster risk in policy implementation. In selecting participants, senior managers and specialists in disaster risk management in Zimbabwe were purposively selected from government departments, non-governmental organisations (NGOs) and universities. These participants were selected from about 40 organisations that participate in the national civil protection committee. In total, 15 semi-structured interviews were conducted, which included five officers from four government departments, five programme managers from four NGOs and five academic staff from three universities.

DA was used to investigate the participants’ construction of disaster risk problems and policy responses. DA is useful in examining multiple and conflicting concepts, ideas and narratives that society holds about an issue (Hajer 1995). DA recognises that practitioners, as actors involved in policy implementation processes, give different meanings to ideas and concepts (Fischer 2003). This is in line with this research, as the terms ‘disaster’ and ‘disaster risk’ have provoked contested debate on what they mean (Perry 2007; Quarantelli 2005). For interview data analysis, interview records were transcribed to Microsoft Word documents and were imported into NVivo. This allowed the development of an initial code skeleton, which included the main themes discussed in the interviews. These themes were the following: (1) disaster causality, (2) policy objectives, (3) policy responses and (4) understanding what limits tractability. Analytical coding (Richards 2009) was then performed to generate new categories that formed the sub-themes, which were ordered into NVivo nodes.

## Results and discussion

This section discusses the results from the interviews. The discussion focuses on disaster causality in order to understand policy objectives and the type of policy responses. Attention is given to the factors that affect tractability of the objectivist frame of disaster risk in policy implementation.

### Disaster causality (objectivist frame)

To the majority (80%) of the participants interviewed, the main causal agent responsible for the Tokwe-Mukosi flood disaster was pinned on heavy rainfall. Thus, the disaster was seen as an ‘act of nature’. This construction of the problem is also reflected in the Tokwe-Mukosi Flood Disaster Lessons Learned Workshop opening speech of the Minister of Local Government Public Works and National Housing: ‘… the flood disaster was due to high precipitation that resulted in the unanticipated rapid filling up of the Tokwe-Mukosi dam that was still under construction’ (Department of Civil Protection 2014:16). Such framing of the problem exemplifies the objectivist view of disasters where causality is placed on the hazard. In line with Lavell and Maskrey (2013), disasters are still viewed as exogenous, unexpected, extreme events that randomly affect otherwise ‘normally’ developing societies. This construction is perhaps not surprising because the *Civil Protection Act* 2001 (Part I, Section 2), which governs disaster management in Zimbabwe, places more emphasis on the hazard (Chipangura et al. 2017). The *Civil Protection Act*’s way of framing disaster risk influences participants’ sense-making about disasters, providing them with an interpretive frame through which they construct their understanding of disasters (Coburn 2006). Thus, the Act’s framing of the problem as an ‘act of nature’ carries more weight in the problem-framing process than frames put forth in counter-framing such as the vulnerability frame.

However, the validity of this causal theory has been criticised by authors such as Van Niekerk (2012) and Pelling (2003) who state that disaster and disaster risk cannot be defined by the hazard component alone. Instead, disaster and disaster risk derive from a combination of hazards and the vulnerabilities of exposed elements. As Cardona (2003) argues, one cannot be vulnerable if one is not threatened, and one cannot be threatened if one is not exposed and vulnerable. Thus, framing disaster risk problems using the objectivist perspective alone presents a partial view that will lead to partial solutions to disaster risk management (Chipangura et al. 2017). Participants who were more inclined to the constructivist perspective of disaster risk found this shortcoming of the hazard paradigm as a contributing factor to the Tokwe-Mukosi flood disaster. An interviewee from a local NGO had this to say: ‘… the problem is that the hazard approach being used to manage disasters does not address vulnerability issues which are critical in causing disasters …’ The key point to recognise here is that the objectivist perspective by ignoring the social construction view of disasters can mask the true roots of disaster risk problem and disaster risk creation activities. Thus, without appropriate theoretical basis and theoretical validity a policy will give wrong directions in all ways (Khan 2016; Sabatier & Mazmanian 1980). This is because policy objectives as presented below are coined to deal with what is perceived to be the problem.

### Policy objectives

As Handmer and Dovers (2013) argue, the way in which a problem is defined and framed circumscribes the search for solution to that problem. By defining the problem as an ‘act of nature’, about 73% of the interviewees argued that public policy should be designed to prepare people and organise reactions to the disastrous effects of hazards such as floods. In this way, 67% of the interviewees stated that the government and NGOs would be able to intervene before, during and after the impact of a hazard. Disaster risk policy objectives are therefore centred on improving disaster preparedness and response. This is supported by a male interviewee who participates in the National Civil Protection Coordination Committee (NCPCC), who stated that ‘… policy must improve disaster preparedness and response …’ Furthermore, this objective is reflected in the Service Charter of the Department of Civil Protection, which puts emphasis on emergency preparedness. The interviews also revealed that these objectives are influenced by the assumption that ‘nature’ is to blame for disasters and reaction to disasters is regarded a battle between ‘nature’ and human beings. Because nature is difficult to predict, human beings must therefore be prepared to respond to disasters. Terms such as ‘civil defence’ and ‘civil protection’ have thus been used in support of this notion in Zimbabwe disaster risk management system. However, this objective fosters a reactionary approach to disaster risk management (Lowe et al. 2007). This means that even if risks are known, authorities tend to wait in anticipation of a disastrous event and then activate plans and procedures. This reactive system is prone to ‘policy surprise’. Policy surprise can be conceived of as lack of preparedness based on erroneous assumptions of whether, when, where and how severely a community might be impacted by a hazard event (Parker et al. 2009). For example, an event can be contrary to the policymakers’ expectations – that is, the initial strategic plan for the Tokwe-Mukosi dam was based on recurrent droughts and engineers believed that the dam would start filling up at a later stage when the communities at risk would have been trans-located (Department of Civil Protection 2014). This assumption was challenged when the southern part of Masvingo region where Tokwe-Mukosi dam is located got more rains than previously expected.

### Policy responses

From the interviews, the policy objective discussed above ‘to improve disaster preparedness and response’ was thought to be achieved through four policy responses: (1) coordination, (2) hazard analysis, (3) improvement of early warning systems and (4) evacuation. These proposed policy responses are in line with Hewitt (1983), who argues that the objectivist frame seeks to manage risk in three ways: (1) anticipate and hence contain the extremes of nature through environmental engineering works, (2) monitor and model extreme geophysical events and (3) create disaster plans and emergency responses. This suggests that as with policy formation, the way in which disaster risk problem is framed plays an important role in how policy implementation unfolds. The four policy responses from the interviewees highlighted above are elaborated below.

#### Coordination

As disaster risk management involves a number of activities and organisations, about 80% of the interviewees stated that coordination within and between disaster risk management organisations is essential to avoid friction and lack of cooperation. In agreement with OCHA (2012), the interviewees revealed that coordination reduces duplication and competition, allowing for complementarity and for scarce resources to be used more effectively to reach more people and fill specific gaps in response to needs. In the Tokwe-Mukosi flood disaster, lack of coordination was seen as an obstacle that needed to be overcome in order to sincerely improve disaster preparedness and response. Three interviewees (two from government departments and one from an NGO) who participate in the NCPCC stated that once coordination is achieved, ‘civil protection’ would take place and would make disaster preparedness and response more effective and efficient. In Zimbabwe, at national level, the execution of the coordination mandate is realised through the NCPCC. Provincial Civil Protection Coordination committees and District Civil Protection Coordination committees are also mandated to coordinate any emergency-related activities in their respective provinces and districts. This ‘top-down view’ normally associated with the objectivist perspective of disaster risk has long been dominant in the study of coordination (Bardach 2001).

However, in the Tokwe-Mukosi flood disaster, effective coordination was difficult to achieve because of the problems associated with the top-down approach to coordination. The results of the interviews revealed that the top-down perspective of coordination by formal organisations fails to adapt to changed conditions in good time. A participant from an NGO interviewed for this research said that: ‘… in rapid onset disasters like this one [Tokwe-Mukosi flood disaster], formal structures and planned responses are too slow, inadequate, and incoherent …’ When one looks at the definition of ‘disaster’, it is not difficult to understand the gist of this argument. Disasters depict a situation in which normal institutions are disrupted because of capacity deficit. This disruption creates an initial confusion as to how to realign plans to the current situation. Thus, modifications necessary for coping with changed conditions are likely to be constrained by the rigidity of the system. The alternative view as suggested by a lecturer from a local university interviewed for this research would be ‘bottom-up’ coordination. He argued that, disasters bring unexpected uncertainty and people on the ground seem to understand the situation better. ‘Bottom-up’ coordination can thus be conceived of as an outcome of local people working together to solve complex disaster risk problems without guidance from the top (Beck & Plowman 2014). Authors such as Faraj and Xiao (2006) and Chisholm (1989) argue that groups in crisis situations work together quite effectively without being guided from the top. The ‘bottom-up’ coordination seems to support the idea of community participation in disaster risk management. An interviewee from a government department who participates in the NCPCC had this to say: ‘… participation in coordination, it is hoped, increases the success of policy and management, because it is inclusive …’ However, this raises a fundamental question: how does bottom-up coordination emerge, and how is it managed?

Sixty-seven percent of interviewees in this research stated that coordination was difficult to achieve because of the diverse composition of people and agencies working together, all of whom possess different skills, knowledge and competencies. The results from the interviews also indicate that emergency agencies sometimes lack a sufficient understanding of the responsibilities, needs, plans and tactics of their own and other participating agencies, which can have a negative impact on coordination. An interviewee from an NGO had this to say: ‘… I do not think responders share a common view on how to respond to disasters like the Tokwe-Mukosi …’ A lecturer from a local university stated that this problem emanates from the lack of an emergency operations plan. According to Coppola (2007), an emergency operations plan clearly describes the people and agencies involved in response to hazards, responsibilities and actions of these individuals and agencies, and when and where these responsibilities and actions will be called upon. The emergency operation plan should be cascaded to all government departments so that all work is organised towards the same goal. Without an emergency operations document, one can argue that it is difficult to deliver a well-coordinated disaster response. Another interviewee from a humanitarian organisation stated that lack of emergency exercises contributed to difficulties in coordination. He had this to say: ‘… pre-disaster preparedness is generally weak because organisations that deal with disasters are not doing enough emergency exercises …’ According to Schwab, Brower and Hoboken (2007), adequate preparedness depends on solid training and exercises that mimic real-life emergency scenarios in controlled setting. This gives practical capabilities and allows individuals to practice their roles and responsibilities before the actual event occurs. Exercises also introduce various individuals and agencies together. As Coppola (2007) argues, disaster management officials will know other disaster responders, understand their roles and responsibility, and find ways of how they can assist and be assisted by others. Thus, as Sabatier and Mazmanian (1980) argue, one of the most important attributes of any statute is the extent to which it hierarchically integrates the implementing agencies.

#### Hazard analysis

Sixty percent of the interviewees in this research stated that hazard analysis was crucial in providing protection to communities. For example, a male interviewee from a government department who participates in the NCPCC said that: ‘… if you do not know the hazards and their potential impact … how can you manage them?’ A hazard analysis involves knowledge of the kinds of hazards that might threaten the community. This knowledge includes the probability of the event occurring at varying levels of intensity and at varying locations in a community (McLoughlin 1985). In Zimbabwe, the *Water Act* Chapter 20:24, which regulates dam safety and construction, puts emphasis on hazard analysis. It categorises dams into four classes which are related to the hazard potential based on the risk to life and the economic consequences that can arise if the dam fails. Class 1 dams are those that give the worst-case scenario and are designed to the highest standards. According to the Act, it is mandatory for dam owners to inform the Ministry of Environment, Water and Climate of possible or existing threat of flooding and taking appropriate measures to prevent and control flooding from such dams. Tokwe-Mukosi dam whose wall height is 89.2 m, and which has a capacity of 1802, 600 000 m^3^, is categorised as class 1 dam (Department of Civil Protection 2014). Results of the interviews also showed that hazard analysis was believed to be crucial because it provides information about the consequences of a possible dam break which is essential in risk estimation and evacuation planning.

However, in the Tokwe-Mukosi flooding, tractability of hazard analysis was limited as few outputs could be produced to avert the flood disaster. Limiting factors cited by interviewees to explain this situation were linked to lack of methodologies, frameworks and software tools necessary to proactively manage flood wave propagation, breaching of embankments and dam break sediment effects. An engineer from a local university who was interviewed for this research stated that the existing flood risk analysis hardly addresses the problems of working with rare hydrological events (*records of river flow rarely exceed 100 years, yet typical design standards of 200 years are required*). This is also reflected in the Tokwe-Mukosi Flood Disaster Lessons Learned Workshop, where a Zimbabwe National Water Authority (ZINWA) representative stated that ‘… inflows into the Tokwe-Mukosi dam were not predictable and much of what took place in terms of the emergency evacuation was not planned for …’ A male project officer from an NGO also said that:

‘… it is difficult to estimate the consequences that will result from the actual flooding, and the probabilities of the success of actions such as evacuation of vulnerable people like the elderly …’ (Project officer from an NGO, male)

In line with views of Kwon and Moon (2005), the distributions used to model some of the random variables are inappropriate relative to the expected behaviour of these variables, and as a result, simulations of the system can lead to unrealistic values of extreme rainfall or water surface levels, and hence of the probability of dam overtopping. The availability of technologies to solve the problem is therefore crucial in improving the tractability of hazard analysis.

Lack of financial resources was cited by about 40% of the interviewees as a limiting factor to achieve a comprehensive hazard analysis. An interviewee from a government department stated that ‘… funds for disasters are insufficient …’ Hazard analysis requires sophisticated and expensive equipment for modelling and highly trained personnel. Money is thus obviously necessary to buy equipment and to hire staff in order to conduct the technical analysis involved in the hazard analysis. An engineer from a local university said that lack of funding has the potential to disrupt or delay policy implementation. In general, a threshold level of funding is necessary for there to be any possibility of achieving statutory objectives (Sabatier & Mazmanian 1980). Participants interviewed, especially from NGOs, also blamed the technocratic oriented hazard analysis for ignoring community participation. An interviewee from a local NGO had this to say: ‘… hazard analysis uses scientific jargon which many locals cannot understand and as such it is difficult to engage them’. As Peters (2005) argued, high levels of technical content can create obstacles for community participation. Thus, even if citizens have opinions, they are unlikely to be effective participants in the process unless they have substantial technical expertise (Peters 2005). One can argue that the scientific and technical hazard analyses give authority to experts who then define the ‘feasible’ course of action.

#### Improvement of early warning systems

As discussed above, objectivism is based on the belief that the hazard is the causal agent of disasters and as such human beings must be prepared to respond to disasters. Sixty percent of the interviewees responded that to improve disaster preparedness and response, a well-functioning early warning system that can deliver accurate, reliable and understandable warnings, in a timely manner, to disaster risk practitioners and populations at risk is crucial. As heavy rains were blamed for the disaster, early warning systems were skewed towards weather-related issues. The need for an early warning system is emphasised in the *Meteorological Services Act*, the National Climate Change Response Strategy and the Department of Civil Protection’s Service Charter. Early warnings and weather forecasts are given by the Meteorological Services Department (MSD). This information is used in forecasting the river flows by ZINWA so as to assess whether there will be floods. Based on this, the appropriate authorities take the necessary steps to ensure that information is disseminated and the potential victims are evacuated before or during the flood events. The rationale for using early warning was believed to increase accuracy, lead time, communication and dissemination of severe weather and flash flood warnings to communities.

However, the tractability of early warning systems was found to be affected by how various components of the system feed into each other. As Jubach and Tokar (2016) argue, early warning systems are truly end-to-end in nature, that is, they consist of a warning and response system where the components are interconnected. Each component in this process is critical in reducing the impacts of hazards and provides essential lead times to aid decisions. Failure of one component will lead to the failure of the entire system to save lives and livelihoods (Jubach & Tokar 2016). In the interviews in this research, two problems were noted. The first problem was the inadequate lead time between the flood forecast and the flood event. The interviews revealed that this is because the models that are being used for meteorological forecasts only provide very short forecasts in an accurate manner. According to the Department of Civil Protection (2014), ZINWA had no capacity for flood forecasting and modelling and would need to investigate the integration of these in its systems. In a tight coupling system, the adverse impacts of a failure in one system may propagate, and possibly amplify, through a number of other connected systems (Vespignani 2010). The second problem was failure to package scientific and technological information in understandable formats by communities. This made it difficult to provide accurate information on the risk associated with flooding to communities downstream. Participants from NGOs interviewed for this research stated that scientific and technical jargon systematically limits community participation as communities find it hard to understand. An interviewee from a government department had this to say: ‘… the majority of downstream residents were not aware of the extent of the flooding that could result from a dam failure’. Arguably, even state-of-the-art technology and a perfect forecast will not save lives if the populations at risk are not informed in a timely manner or do not understand the message. Consequently, well-prepared communities remain vulnerable to hazards if they do not have access to and understand information that provides the lead time needed to take necessary actions. Communications and warning or information dissemination are important attributes of a successful warning system.

#### Evacuation and rescue operations

On evacuation, approximately 53% of the interviewees of this research felt that evacuation of potential flood victims and vulnerable property when accompanied by advance planning, warning and response, and subsequent sheltering is an important means of reducing loss of life. Evacuation involves people moving from their houses or places of business to ‘safe’ locations, out of the flood risk area where they are able to shelter until it is possible and appropriate for them to return. Two interviewees from the NCPCC were convinced that evacuation before the arrival of flood must be the main approach as it ensures safety of people and property. This implies that there should be adequate arrangements of where to relocate the displaced people including the provision of food, clothing and sanitation. This view was believed to be based on the idea of ‘civil protection’.

However, evacuation exercise was burdened by a number of challenges. According to the Department of Civil Protection (2014), these challenges included lack of funds and resistance by some of the families that were supposed to be relocated. The results of the interviews revealed that the public was not aware of evacuation routes, safe locations and warning methods. One participant from an NGO in her 40s stated that:

‘… in this area I have not seen or heard of any flood drills being done and I haven’t seen or heard of any contingency plans for evacuation being put into practice by affected residents …’ (Female)

This implies that community participation was neglected. About 27% of the interviewees mentioned that overlooking local people’s capacity and coping strategies was dangerous because it results in conflicts and worsens vulnerabilities and complicates emergency response. Interviewees viewed ineffective evacuation as being more caused by lack of community participation, and not in lack of local coping capacities. Community participation was seen as crucial because it was believed that it improves information flow, fosters collaboration, minimises conflicts and enhances community understanding of evacuation. Poor evacuation was also blamed on the idea of ‘civil protection’. An interviewee from a local university told that the idea of ‘civil protection’ was misleading because the role of the government is seen as that of a protector and the local people are conceived of as passive subjects who simply wait to receive information and directions from the government through the civil protection sector in disaster situations. Communities may thus find comfort in the fallacy of protection. They behave like children who always think older and powerful people will protect them and find comfort in the notion that, ‘if something bad happens to us, someone else bigger and better than us will come to our rescue, absorb our loses, bail us out’ (Mitroff & Pauchant 1990). This undermines people’s own capacities and coping strategies (Pelling 2003).

## Conclusion

From the presentation above, this article clearly shows that ‘framing’ harbours ‘latent’ failures, which only become noticeable on the occurrence of a particular disaster. In the objectivist frame of disaster risk, these ‘latent’ failures are hidden in diagnostic framing, which, in turn, affects its tractability. In its framing of disaster causality, the objectivist frame places much emphasis on natural hazards and tends to ignore socio-economic processes that place the vulnerable populations at risk. Consequently, this frame has tended to foster technocratic and bureaucratic approaches to disaster risk reduction, which then further feed the dominant concepts and imaginaries in a self-reinforcing manner (Lavell & Maskrey 2013). Efforts to minimise the devastating consequences of disasters have therefore been channelled towards dealing with the hazards using science and technology rather than the underlying vulnerabilities, which generate disaster risk. The use of scientific and technical jargon and methodologies has been found to effectively exclude communities from participating in the process of framing the problem, generating options, evaluating options and coming to joint conclusions. Furthermore, the technocratic approach associated with the objectivist frame requires significant administrative and technical expertise and funding to be tackled effectively, which are not readily available especially in developing countries. By closing off the social constructivist door one can argue that framing leads to ‘ignorance’ of rival frames. This ‘ignorance’ is not ignorance in the ordinary sense of not knowing, rather it is knowledge based on erroneous cognitive beliefs. Thus, in managing disasters, frames that threaten the dominant frame will be ignored, reinterpreted, hidden or rejected in a way analogous to the methods that individual resort to defend their own self-esteem (Brown & Starkey 2000).

This research demonstrates that framing shapes how implementation unfolds by opening up some avenues for action while simultaneously closing off others. By this, it sets parameters within which decision-making unfolds, thereby determining the regulatory path and how tractable the chosen strategy is going to be. The objectivist frame therefore becomes prone to ‘policy surprise’, that is, lack of preparedness based on erroneous assumptions of whether, when, where and how severely a community might be impacted by a hazard event (Parker et al. 2009). This can lead to ‘policy disasters’, where disasters are viewed as direct consequences of policy choices. In a tight coupling system with rigid operational structures (which it promotes), the adverse impacts of a failure in one system may propagate, and possibly amplify, through a number of other connected systems causing more chaos. In this condition, modifications necessary for coping with changed conditions are constrained by the rigidity of the system. Thus, technocratic solutions must not only be based on one system of interest, but also the unexpected consequences that cascade through other connected systems. This article thus concludes that for Zimbabwe to achieve the goal of minimising the devastating impacts of disasters, greater efforts must be made in reframing disaster risk by integrating objectivism with constructivism. This is because disaster risk derives from a combination of physical hazards and the vulnerabilities of exposed elements.
